# Emergency right lower lobectomy for severe pulmonary abscess in a pregnant woman at the 25th week of gestation: a case report

**DOI:** 10.1186/s40792-024-01932-8

**Published:** 2024-05-23

**Authors:** Haruaki Hino, Yuki Yasuhara, Katsutoshi Nakahata, Takahiro Utsumi, Natsumi Maru, Hiroshi Matsui, Yohei Taniguchi, Tomohito Saito, Koji Tsuta, Hidetaka Okada, Tomohiro Murakawa

**Affiliations:** 1 Department of Thoracic Surgery, Kansai Medical University, 2-5-1 Shinmachi Hirakata, Osaka, 573-1191 Japan; 2 Department of Obstetrics and Gynecology, Kansai Medical University, Osaka, Japan; 3 Department of Anesthesiology, Kansai Medical University, Osaka, Japan; 4 Department of Pathology, Kansai Medical University, Osaka, Japan

**Keywords:** Pulmonary abscess, Pregnancy, Immune tolerance, Emergency lung resection

## Abstract

**Background:**

Pulmonary abscess is a severe infection commonly seen in patients with chronic obstructive pulmonary disease, interstitial pneumonia, immune deficiency disease, drug-induced immunocompromised state, and congenital pulmonary disease. The treatment strategy in pregnant women with a pulmonary abscess is considered challenging since adverse effects on the fetus must be avoided to ensure safe delivery.

**Case presentation:**

A 34-year-old female patient at 24 weeks of gestation (G2P1) was admitted to the Department of Obstetrics and Gynecology due to sudden right chest pain. The patient had no significant medical history, including congenital anomalies, and no history of drug addiction or smoking. Laboratory data indicated high levels of inflammation (white blood cell 12,000/µL, C-reactive protein 16.0 mg/dL), and computed tomography demonstrated a large intrapulmonary cyst located in the middle of the right lower lobe, with some fluid collection. As the patient had no medical history of congenital pulmonary anomalies, she was initially diagnosed with a pulmonary cyst infection and treated with intravenous antibiotics. However, the infection did not resolve for over a week, and a spike in fever developed after admission. There was no definitive evidence concerning the risk of preterm delivery and fetal abortion during non-obstetric surgery. However, to control the severely infected pulmonary abscess that was refractory to antibiotics and obtain a pathological diagnosis while saving the life of both the mother and fetus, we elected to perform an emergent right lower lobectomy by open thoracotomy with a fissureless maneuver after receiving informed consent. Postoperatively, the infection gradually improved, and the patient was discharged on the 16th postoperative day without any major complications in the mother or fetus. Although she later experienced coronavirus disease-19 at 29 weeks of gestation, a boy was born at 40th weeks of gestation without any complications. Pathologically, no infectious agents, malignancies, or congenital anomalies other than lung abscesses associated with the pulmonary infarction were observed. The mother and child were healthy 1 year postoperatively.

**Conclusions:**

We experienced a rare case of a pulmonary abscess in a pregnant woman who needed an emergent right lower lobectomy to control the severe infection and obtain a correct pathological diagnosis. Under cooperation from an obstetrician and anesthesiologist, emergency pulmonary resection can be performed safely for serious abscess formation even for pregnant women who have several months left until delivery.

## Background

Pulmonary cysts can present in various forms depending on the congenital disease, such as congenital cystic adenomatoid malformation or other acquired disorders, including chronic obstructive pulmonary disease (COPD); interstitial pneumonia; infections, such as coccidioidomycosis and *Pneumocystis carinii*; pulmonary lymphangioleiomyomatosis; and malignancy [1]. Emergency treatment is required in cases of pulmonary cyst infections or abscess formation complicated by bleeding or sepsis. Severe pulmonary abscesses complicated by pregnancy are relatively rare; prompt treatment is required to save the lives of both the mother and fetus [2]. According to a nationwide study in Finland, non-obstetric surgery for pregnant women (*n* = 3619), including gastrointestinal surgery (*n* = 1904, 44%), orthopedic surgery (*n* = 558, 13%), and thoracic surgery (*n* = 22, > 1%), is performed in approximately 0.4% of all pregnancies, making it a relatively rare entity for the Department of General Thoracic Surgery [3]. Herein, we report a case of an emergency right lower lobectomy via thoracotomy that was performed to control a severe pulmonary abscess with a septic condition in a pregnant woman at 24 weeks of gestation.

## Case presentation

**Fig. 1:**
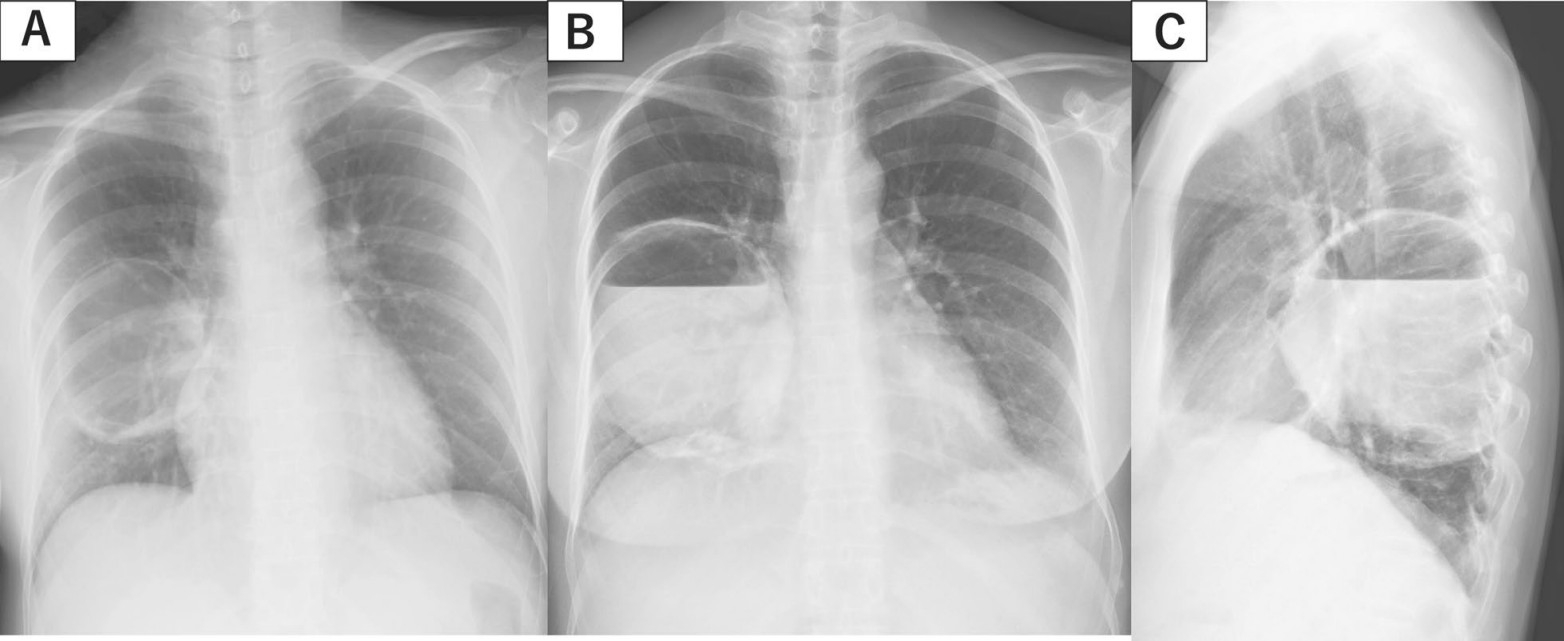
Preoperative chest X-ray. **A** Chest X-ray on admission showing pulmonary cyst formation in the right lower lung field. **B** Front-view chest X-ray at surgery showing massive fluid collection in the pulmonary cyst. **C** Chest X-ray from the side view at the operation also showing massive fluid collection in the right lower lobe

**Fig. 2:**
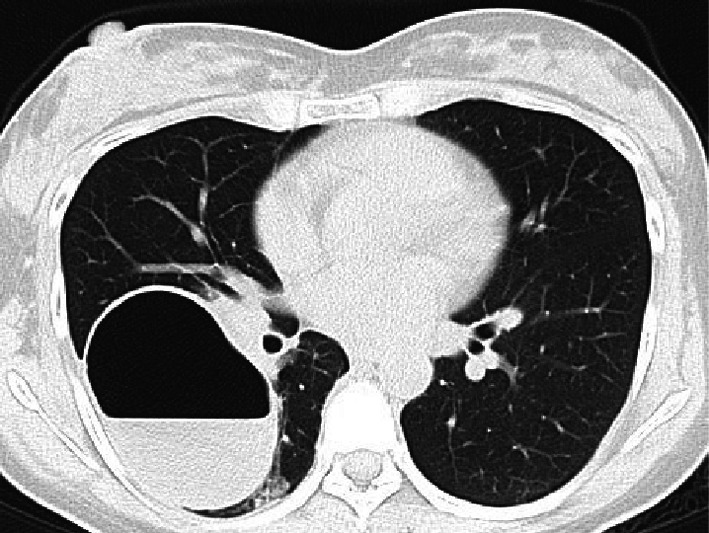
Preoperative computed tomography and postoperative chest X-ray. Chest computed tomography showing a pulmonary cyst with abscess formation in the middle of the right lower lobe

**Fig. 3:**
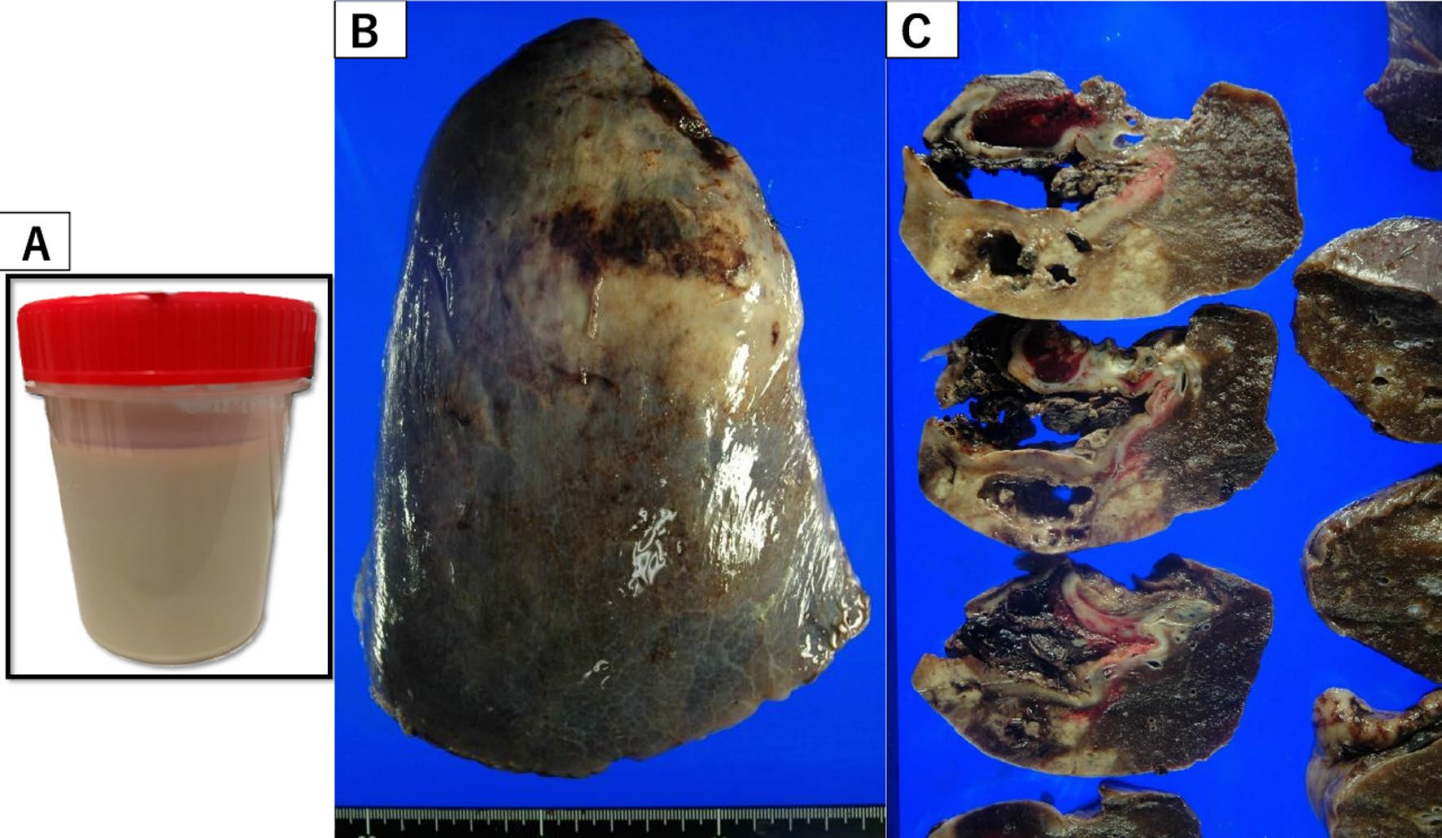
Excised abscess and macroscopic view of the resected lung. **A** Abscess removed during operation of the pulmonary cyst. **B** Entire view of the resected right lower lobe. **C** Cut surface of the resected right lower lobe showing a lung abscess

**Fig. 4:**
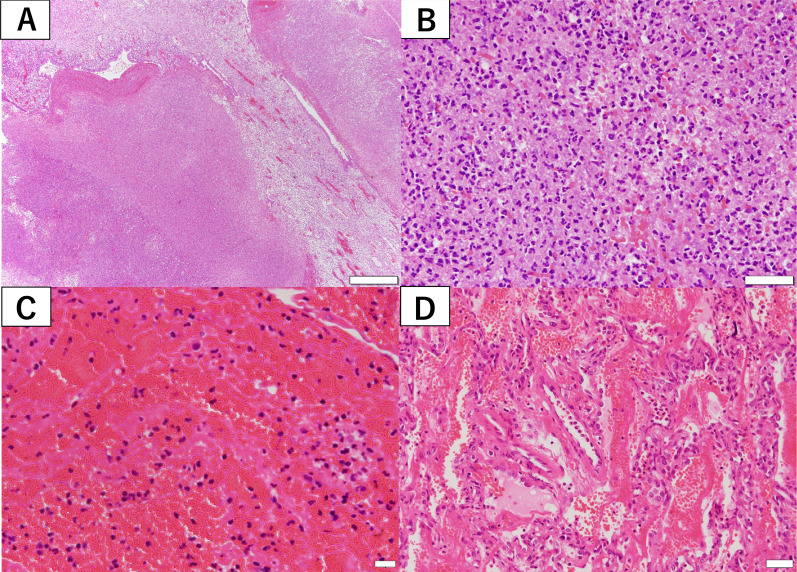
Histopathological examination with Hematoxylin and eosin staining of the resected right lower lobe. **A** Cyst wall complicated with abscess formation in low magnification. Bar = 1 mm. **B** Abscess formation with neutrophil infiltration in high magnification. Bar = 50 μm. **C** Pulmonary infarction with fibrin precipitation detected as a secondary change. Bar = 20 μm. **D** Diffuse alveolar damage detected in obstructive pneumonia around the infected pulmonary cyst. Bar = 50 μm

A 34-year-old female patient at 24 weeks of gestation was admitted to the Department of Obstetrics and Gynecology of our institution due to sudden right chest pain. She had no smoking history or addiction to drugs, such as methamphetamine. She also had previously delivered a female baby at the age of 27 years; however, she had no relevant medical history, such as congenital pulmonary anomalies or a history of undergoing an X-ray at the previous delivery. In the past, at the age of 25 years, she had undergone an X-ray; however, no abnormal changes were detected. Laboratory data at admission showed a white blood cell (WBC) count of 16,000/mL (normal range 4000–9000/mL), C-reactive protein (CRP) level of 15.03 mg/dL (normal range < 0.03 mg/dL), and body temperature of 37.1 °C. Chest X-ray revealed a pulmonary cyst outline in the right lung field at admission (Fig. 1A). Although the origin of the pulmonary cyst was unknown, non-invasive treatment with intravenous antibiotics (sulbactam/ampicillin, 3 g every 8 h) was initially administered in an attempt to control the infection. However, a fever spike of 38 °C was observed 5 days after admission. The antibiotic regimen was subsequently changed to tazobactam/piperacillin (4.5 g every 8 h) due to suspected anaerobic bacteria infection, which was administered for 5 days. Despite antibiotic treatment, the pulmonary cyst continued to gradually grow and was complicated by massive fluid collection, as shown by chest X-ray (Fig. 1B, C) and computed tomography (Fig. 2). Thereafter, she was referred to our department. Conservative management was not effective at all and the patient’s infection was worsening, becoming complicated with pulmonary abscess. Therefore, we planned to perform an emergent right lower lobectomy via open thoracotomy to control the refractory, serious infection with the septic condition and to save the lives of both the mother and the mid-term fetus. In addition, pulmonary resection enabled a histopathological diagnosis to elucidate the etiology of the cyst. Preoperatively, as there was no definitive evidence to suggest a risk of fetal preterm delivery or abortion, informed consent from the patient and her family was obtained. During general anesthesia, the following drugs were administered: propofol (2 mg/kg) for inducing anesthesia (injected intravenously), sevoflurane 1.0–1.2% via inhalation, rocuronium bromide (total; 1.0 mg/kg) and remifentanil hydrochloride (0.1–0.2 μg/kg/min) intravenously, and levobupivacaine hydrochloride injected through an epidural catheter. When the blood pressure was below 80 mmHg, ephedrine hydrochloride (total: 12 mg) and phenylephrine hydrochloride (total: 0.1 mg) were injected temporally. At the end of general anesthesia, sugammadex sodium (total; 200 mg) was used for recovery. Intraoperatively, the right lower lobe of the lung was found to be filled with a massive abscess in the intrapulmonary cyst, which interfered with the surgical field; hence, we initially aspirated the foul-smelling infected fluid as much as possible (Fig. 3A). Subsequently, no interlobar fissure was seen due to the infection and adhesions; therefore, a fissureless lobectomy to resect the inferior pulmonary vein and lower bronchus was performed, and finally, resection of an interlobar fissure and the pulmonary artery was performed simultaneously using linear staples. During resection of the interlobar fissure, the right upper and middle lobes were partially resected for complete excision of the infected cyst. To allow perioperative monitoring of the fetus's heartbeat, a Doppler echo was performed by an obstetrician immediately preoperatively and postoperatively to monitor the heart rate and contractions. The operation time was 176 min with a total blood loss of 386 mL. The chest drain was removed on the 12th postoperative day due to excessive pleural effusion and risk of empyema. The patient was discharged on the 16th postoperative day on foot with controlled inflammatory findings (WBC count, 10,600/mL; CRP level, 1.164 mg/dL). As for postoperative pain relief, oral pain reliever, such as acetaminophen (2 g/day), which do not affect fetal ductus arteriosus closure based on our past experience, was prescribed; moreover, epidural anesthesia with an injection of levobupivacaine hydrochloride was also utilized for postoperative pain control. However, loxoprofen, one of the NSAIDs, which affect ductus arteriosus closure as well as pulmonary hypertension and fetal abortion, was not prescribed. Pathological examination revealed lung abscess with no infectious agents, malignancy, or congenital anomalies, such as congenital cystic adenomatoid malformation; a thick cyst wall, fibrin precipitation, and extensive neutrophils infiltrations with pulmonary infarction as well as obstructive pneumonia and diffuse alveolar damage were also ascertained. Furthermore, a fairly large lung abscess extensively invaded and eroded the normal pulmonary parenchyma; therefore, a possible congenital anomaly might have been destroyed, and consequently, those anomalies might not have been detected in the resected right lower lobe pathologically (Figs. 3B, C, 4A–D).

**Fig. 5:**
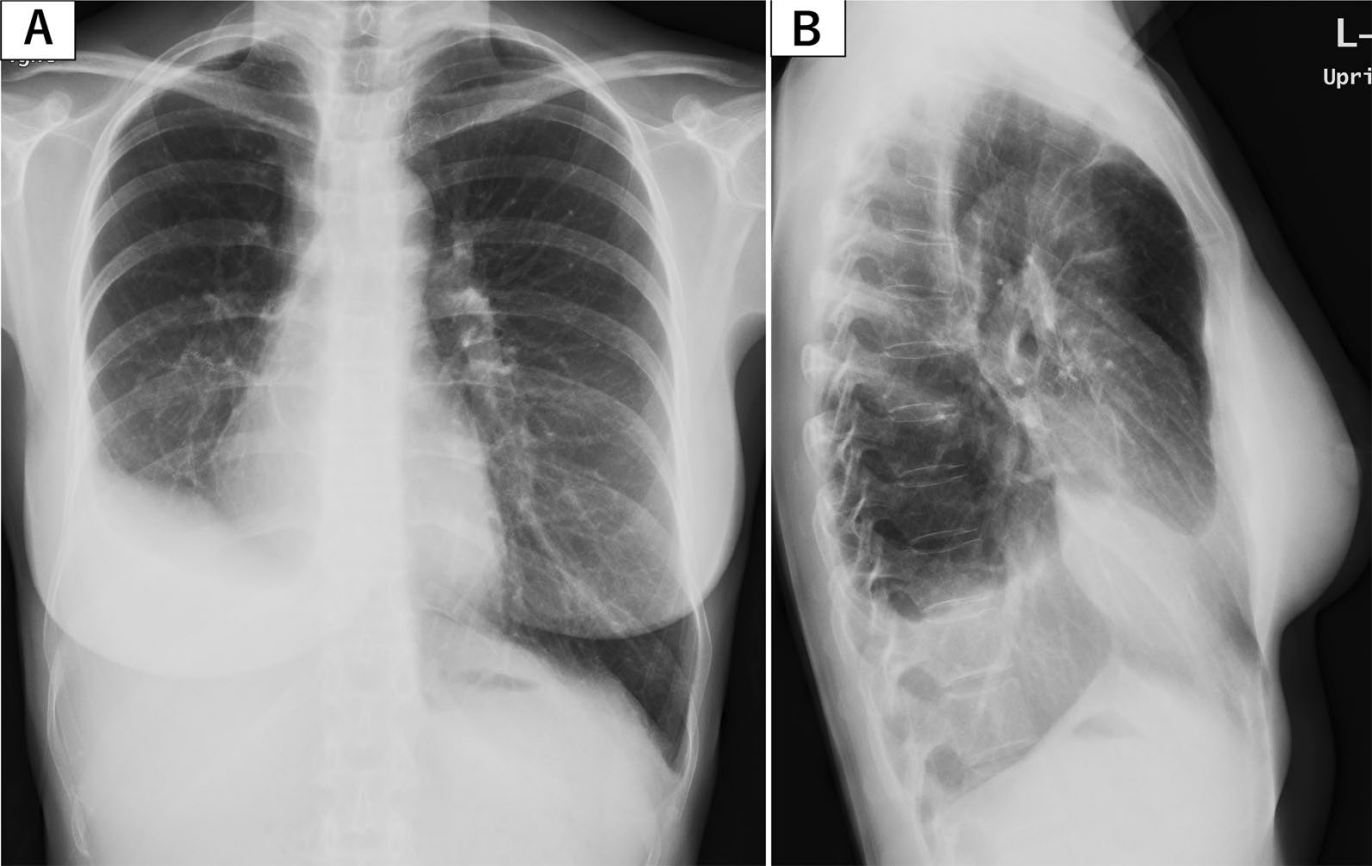
One-year postoperative chest X-ray. **A** Front-view chest X-ray at 1-year after surgery showing normal postoperative pulmonary condition. **B** Side view chest X-ray at 1 year after surgery representing acceptable pulmonary volume

As for the result of the bacterial culture examination, the preoperative sputum culture detected *Moraxella catarrhalis*, α-streptococci, and *Haemophilus* spp.; however, postoperative bacterial culture of the abscess obtained from the right lower lobe intraoperatively revealed only white blood cell (neutrophils), although we suspected infection from anaerobic bacteria based on the foul-smelling. Specific agents, such as bacteria, including anaerobic bacteria, fungus, mycobacterium, and other organisms, were not detected on Gram staining and in bacterial culture tests postoperatively. Polymerase chain reaction tests for *Mycobacterium tuberculosis*, *avium*, and *intracellulare* were also negative. Since sulbactam/ampicillin or tazobactam/piperacillin was administered over 10 days postoperatively, the pathogenic bacteria may have been killed and a microbial substitution phenomenon may have occurred. Considering the pathological and negative bacterial examination, preoperative spike fever with higher inflammatory response might be attributed from peripheral obstructive pneumonia surrounded by large pulmonary cyst. Although the patient later became infected with coronavirus-19 at 29 weeks of gestation, her condition was not serious, and she delivered a male newborn uneventfully at 40 week gestation. One year postoperatively, both the mother and infant were living in healthy condition and chest X-ray showed an acceptable recovery status (Fig. 5A, B).

## Discussion

Pulmonary abscess is a serious infection in patients with COPD, interstitial pneumonia, and congenital or acquired pulmonary cystic disease. Herein, we encountered a pregnant woman with an irreversible severe pulmonary abscess with a septic condition that eroded the lung parenchyma, which was life-threatening for both the mother and fetus. We performed a right lower lobectomy to control this serious infection, subsequently allowing safe delivery of the newborn. Regarding the indications of non-obstetric surgery in pregnancy, the recent guidelines of the American College of Obstetricians and Gynecologists Committee proposed that pregnant women should not be denied surgical treatment considered medically necessary if delayed surgery could adversely affect the health of the pregnant woman and her fetus [4].

Accordingly, an emergency surgery was promptly performed in the present case. However, past publications demonstrated that the preterm delivery rate after non-obstetric surgery was 9.1–9.4%, twofold higher than that of pregnancies without surgery [3]. We considered the balance between the infected general condition and surgical risk management preoperatively and ultimately elected to perform emergency surgery after receiving informed consent. Still, unknown perioperative risks even in mid-term pregnancy were presented, and conservative interventional management of abscess drainage was another therapeutic option for reducing the surgical risk and burden. However, we performed surgery not only to control the prolonged infection but also to obtain a pathological diagnosis. As non-obstetric surgery in pregnant women is relatively rare, especially in general thoracic surgery, surgical timing, procedure, and perioperative management, should be decided with the full cooperation of gynecologists and anesthesiologists along with monitoring the intraoperative fetal heartbeat.

We discussed the perioperative management preoperatively with anesthesiologists and obstetricians. In general, anesthetic agents, including inhaled drugs, such as sevoflurane and fentanyl, can be safely used in pregnant women without any significant side effects on the fetus before and after delivery based on our past experience, although the developing brain is negatively affected to some degree temporally. Especially among anesthetic agents, narcotic drugs, such as fentanyl, and local anesthesia injected epidurally, which have fewer effects on the developing brain, are relatively more feasible for pregnant women and fetuses than other inhaled gases [5, 6]. Therefore, the anesthesiologist performed general anesthesia using fentanyl and epidural anesthesia on a propriety basis. Furthermore, we discussed that a left lateral maternal position intraoperatively was feasible for the patients; however, during anesthesia induction, a dorsal position should be cautiously used because of the risk of hypotension due to the mechanical burden on the venous return from gravid uterus at term. In this case, at the 25th week of gestation, the vena caval compression was not evident, and therefore, the perioperative position was not critical. Apart from the position, the fetal heartbeat should be monitored as an important part of perioperative management, and we co-operated with the obstetrician for the same. Safe management for such patients during pregnancy individually may clarify trustworthy treatment strategies in the future.

Preoperative and postoperative antibiotics selection is important, especially during pregnancy. Basically, antibiotics such as tetracyclines and aminoglycosides should not be used during pregnancy due to the risk of fetal central nervous system disorder. However, penicillin and cephem are known to be safe for the mother and fetus. In this case, an anaerobic bacterial infection was highly suspected, since the location of the infection was within the pulmonary cyst. Therefore, we used intravenous antibiotics, namely, sulbactam/ampicillin, first, then changed to tazobactam/piperacillin, which are more targeted for anaerobic bacterial infection until the end of the operation. Intraoperatively, a foul-smelling infected lung abscess was present. Hence, tazobactam/piperacillin was continued postoperatively until CRP level was under 5 mg/dL. Subsequently, amoxicillin was prescribed orally for 2 weeks until the blood markers indicating inflammation normalized. Though antibiotics were administered in the present case, no drug-induced side effects were observed; however, we should be cautious about any complications related to antibiotics in pregnant women.

As for the surgical approach, first, we thought that the video-assisted thoracoscopic approach was feasible, because a minimally invasive approach is assumed suitable for both maternal and fetal bodies. However, the right lower lobe was probably not deflated, since it was hard and firm even after intraoperative fluid drainage. We also considered that an interlobar fissure might have adhesions due to infection and inflammatory reaction; resultantly, the pulmonary artery and bronchus may not be visible from the interlobar fissure. Furthermore, the duration of general anesthesia should be shortened to minimize the burden in the fetus. Considering the above, we opted for open thoracotomy, instead of a minimally invasive approach to proceed with the emergency right lower lobectomy with a fissureless maneuver with minimal anesthesia, surgical time, and hemorrhage. Resultantly, we performed all the procedures in an acceptable operation time without any postoperative complications in this case. An appropriate surgical approach for pregnant women should be opted for while considering the risks and benefits for the mother and fetus.

The risk of developing a pulmonary abscess is high in immunocompromised patients and in those with an underlying pulmonary disease or an iatrogenic origin [1]. Preoperatively, we considered the possibility that the patient had a congenital lung disease, such as cystic adenomatoid malformation [7], malignancy [8], or other lung diseases of unknown origin. However, close pathological examination revealed no obvious congenital cystic disease, malignant cells, or bacterial or fungal pathogens. Due to the severe destruction of the lung parenchyma by abscess formation, a congenital anomaly or the correct origin of the pulmonary cyst might be obscured. Furthermore, we did not determine the etiology of the irreversible severe pulmonary abscess formation in the patient, who was a young woman with a normal immune status. We speculated that the severe deterioration of the pulmonary abscess with a septic condition may be attributed to specific immunotolerance during pregnancy [9, 10]. Prior research has indicated that pregnant women experience immunodeficiency, which prevents immunologic reactions toward the fetal body itself from the middle of the pregnancy term. These unique immune networks may make cyst infection more serious and, as a result, place maternal and fetal life at risk. Initially, we attempted conservative therapy with antibiotics; however, gradual deterioration of infection was observed for 10 days. Prompt response of emergent surgical lobectomy was subsequently performed, saving the mother and fetus without any serious complications. At the time of the operation, the patient was in the mid-term of pregnancy (24 weeks), and general anesthesia was considered relatively safe for the fetal body. Nevertheless, preoperative informed consent was carefully obtained after explaining the risks of preterm delivery or abortion. In a previous similar case report, a 25-year-old pregnant woman at 25th weeks of gestation presented with a lung abscess from *Streptococcus intermedius* infection which was successfully treated with antibiotics [11]. Another 28-year-old pregnant woman at 22 week gestation had a lung abscess with empyema and was treated by intravenous antibiotics and chest drainage safely without surgery [2]. In contrast, there have been no prior reports of a patient who underwent emergent pulmonary resection for an irreversible severe pulmonary abscess, subsequently allowing safe delivery of a healthy newborn. Therefore, we should carefully consider a balance of risk and benefit for performing lung resection for pregnant women who require it after receiving fully informed consent.

During the preoperative meeting with obstetric physicians, it was noted that a postoperative hypoxic condition might interfere with the healthy development process of the fetus and increase the rate of premature complications by up to approximately 9% [3]. Under the condition of normal pregnancy in both the maternal and fetal bodies prior to lung abscess formation, we performed lung resection not only to control severe infection but also to save the lives of both the mother and fetus. As a result, appropriate judgment, optimal procedures, and postoperative management led to a successful postoperative course cooperating with the obstetrician and anesthesiologist. Initially, we considered chest drainage for abscesses under local anesthesia as conservative management; however, the residual pregnancy term was over 10 weeks at that time, and an investigation for abscess formation in such a normal pregnancy to resolve severely infected conditions via a definitive curable procedure as soon as possible was mandatory. Therefore, the patient was counseled to undergo emergency lung resection, despite the perioperative risk of maternal and fetal life. Fortunately, prompt management by an obstetrician and anesthesiologist contributed to successful results. As the treatment course for a lung abscess in a pregnant woman is rarely reported, this case presentation may help clinicians manage similar cases in the future.

## Conclusion

Herein, we encountered an unusual case of a pregnant woman who presented with a severe, irreversible lung abscess. Emergency lung resection, performed promptly in cooperation with obstetric physicians and anesthesiologists, should be considered to control infection and obtain a pathological diagnosis to save both maternal and fetal lives.

## Data Availability

The datasets supporting the conclusions of this article are available on reasonable request.

## Abbreviations

COPD: Chronic obstructive pulmonary disease
CRP: C-reactive protein
CT: Computed tomography
WBC: White blood cell
